# Supplementary figures and images for: The USH3A causative gene clarin1 functions in Müller glia to maintain retinal photoreceptors
Source: PLoS Genet. 2025 Mar 11;21(3):e1011205. doi: 10.1371/journal.pgen.1011205 (PMC11925288; doi:10.1371/journal.pgen.1011205)

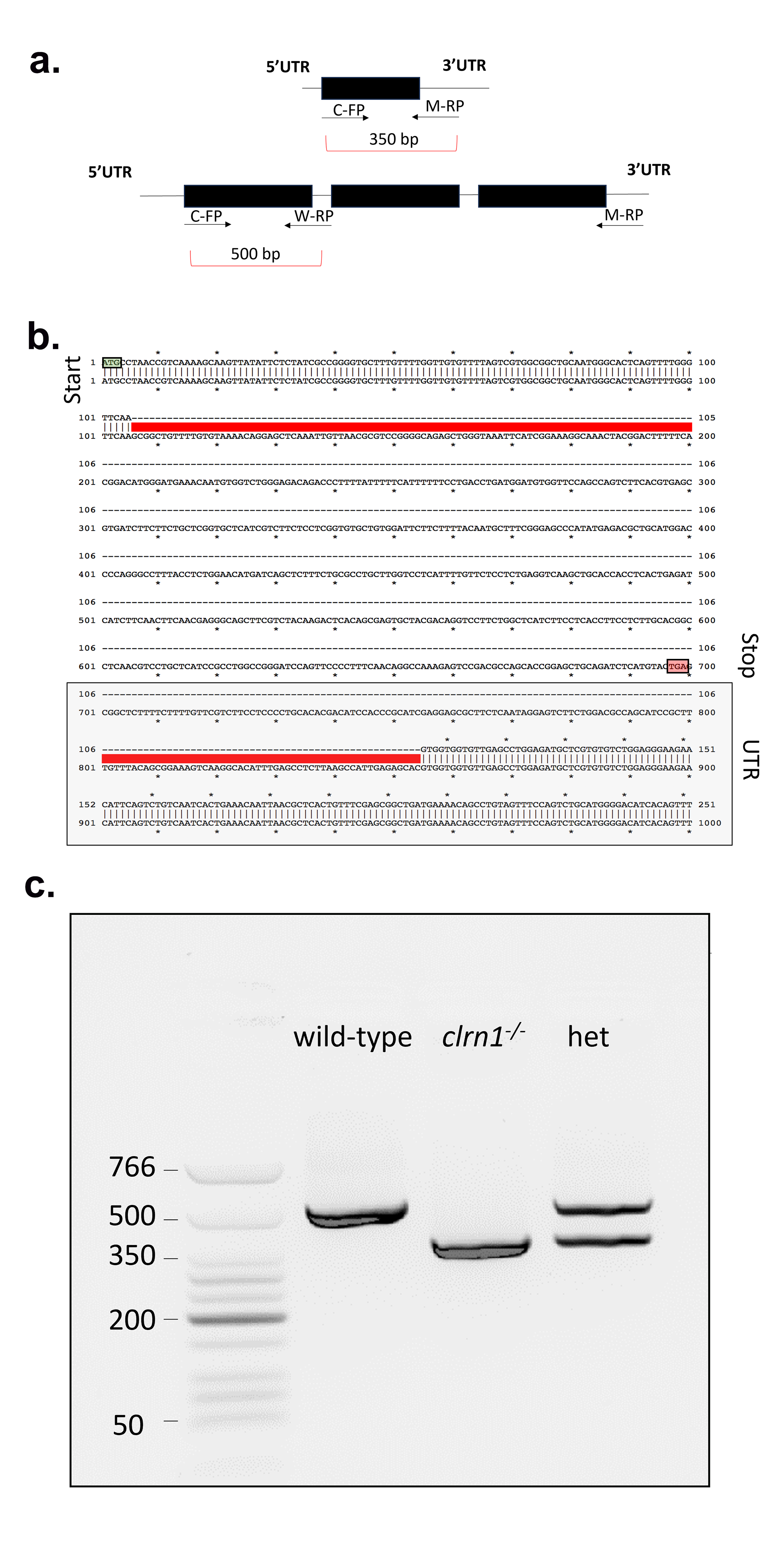

Supplement: S1 Fig — (a) Overview of genotyping assay to identify wild-type, clrn1+/-, and clrn1-/- zebrafish. A common forward primer (C-FP) was designed upstream of the exon 1 cut site, while a mutant reverse primer (M-RP) was designed downstream of the 3’ UTR cut site, and wild-type reverse primer (W-RP) was designed internal to the exon 1 cut site. (b) Generation of the large deletion in the clrn1 coding sequence was confirmed with Sanger sequencing. (c) Example gel depicting the PCR amplification products for wild-type, clrn1-/-, and clrn1+/- (het) zebrafish. (TIFF) [file pgen.1011205.s001.tif]

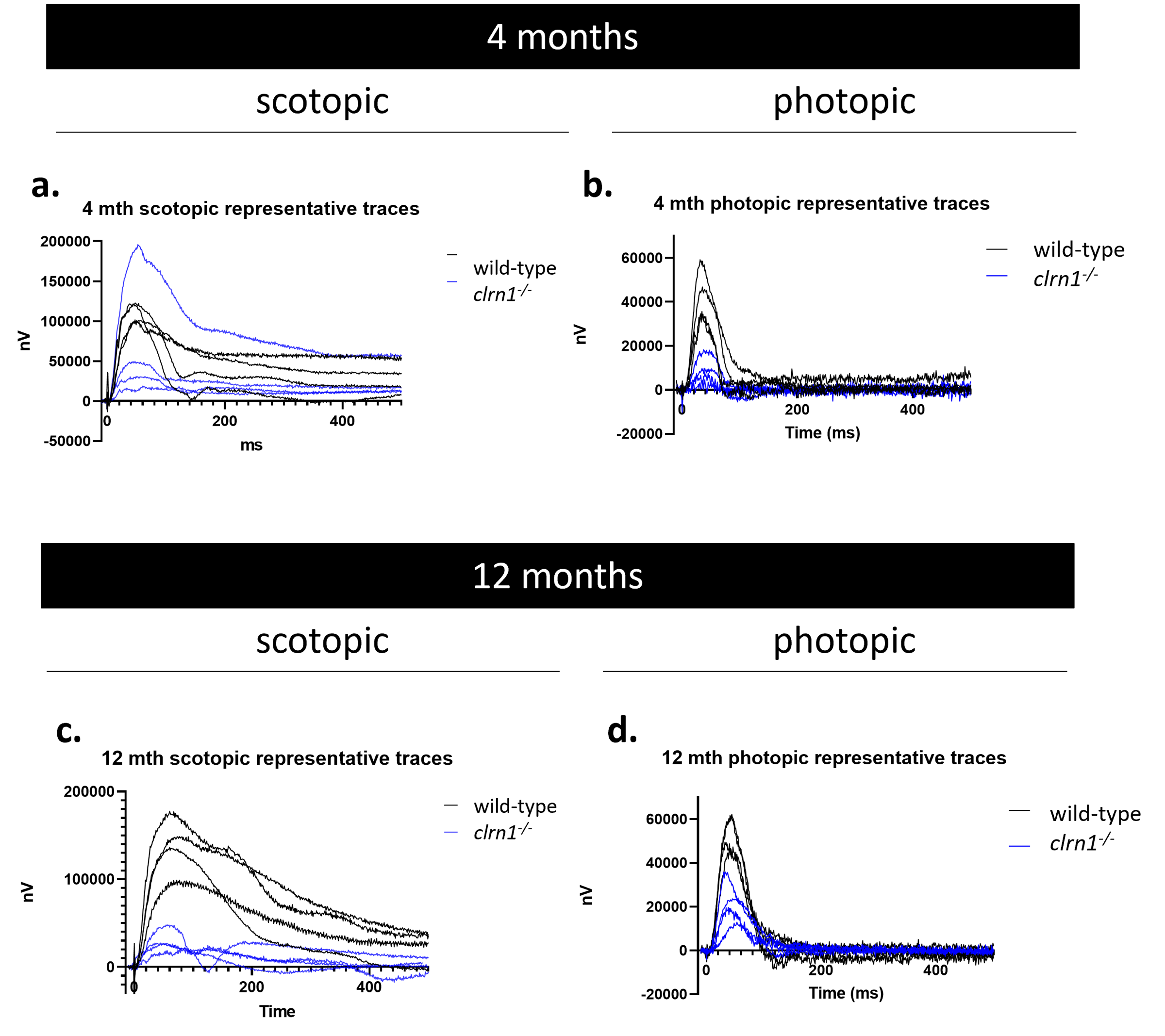

Supplement: S2 Fig — Representative traces from four 4 mpf wild-type (black traces) and clrn1-/-(blue traces) (a) scotopic and (b) photopic responses to highlight high variability in the clrn1-/- scotopic b-wave. Representative traces from 12 mpf wild-type and clrn1-/- (c) scotopic and (d) photopic responses. (TIFF) [file pgen.1011205.s002.tif]

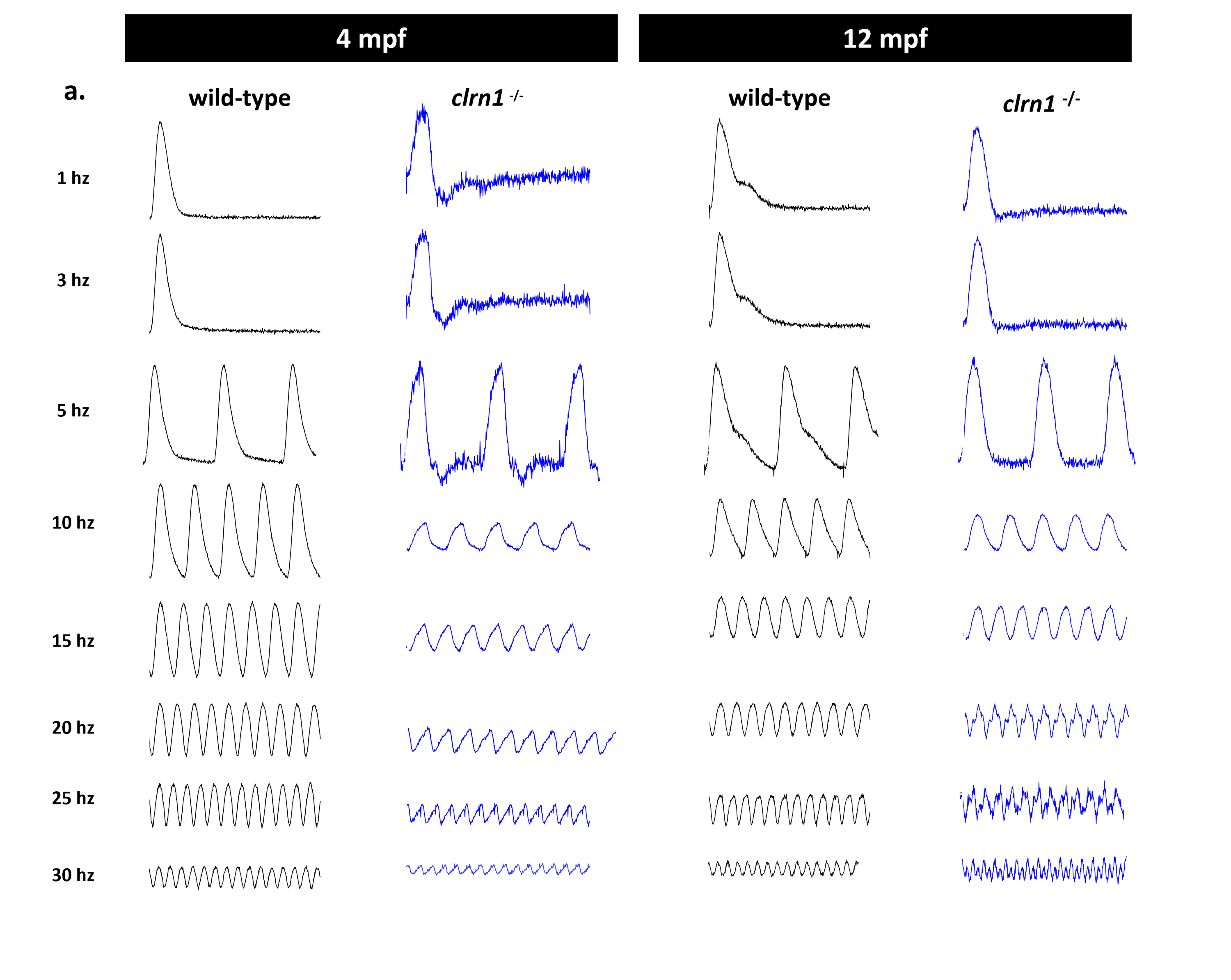

Supplement: S3 Fig — Representative photopic flicker traces of 4 and 12 mpf wild-type (black traces) and clrn1-/- (blue traces) at a range of 1-30 hz. (TIFF) [file pgen.1011205.s003.tif]

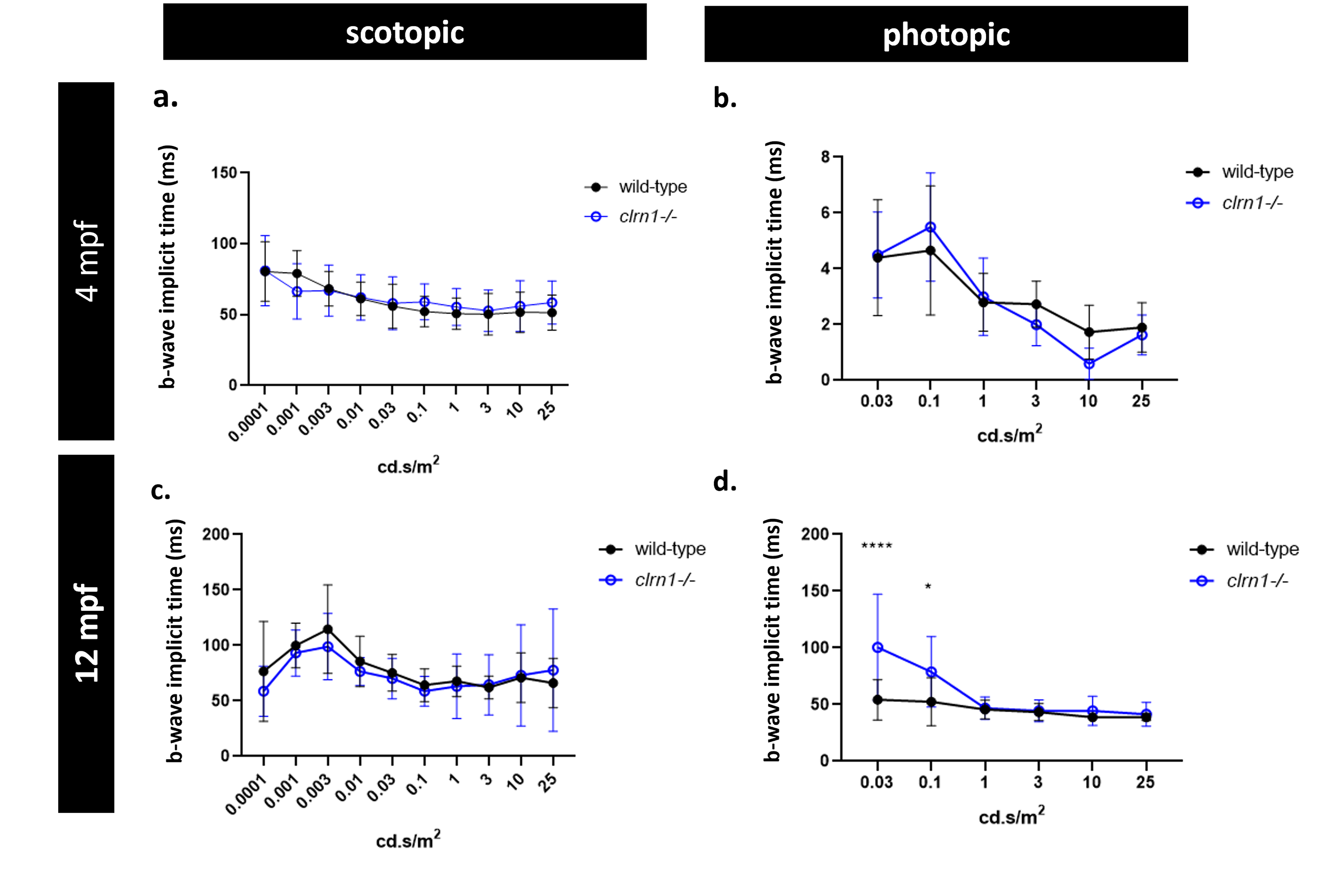

Supplement: S4 Fig — (a) Scotopic and (c) photopic b-wave implicit time for wild-type and clrn1-/- zebrafish at 4 mpf. (c) Scotopic and (d) photopic b-wave implicit time for wild-type and clrn1-/- zebrafish at 12 mpf. (*p<0.05, ****p<0.001; One-way ANOVA) (n=10 animals/eyes per genotype for each time-point). Error bars=SD. (TIFF) [file pgen.1011205.s004.tif]

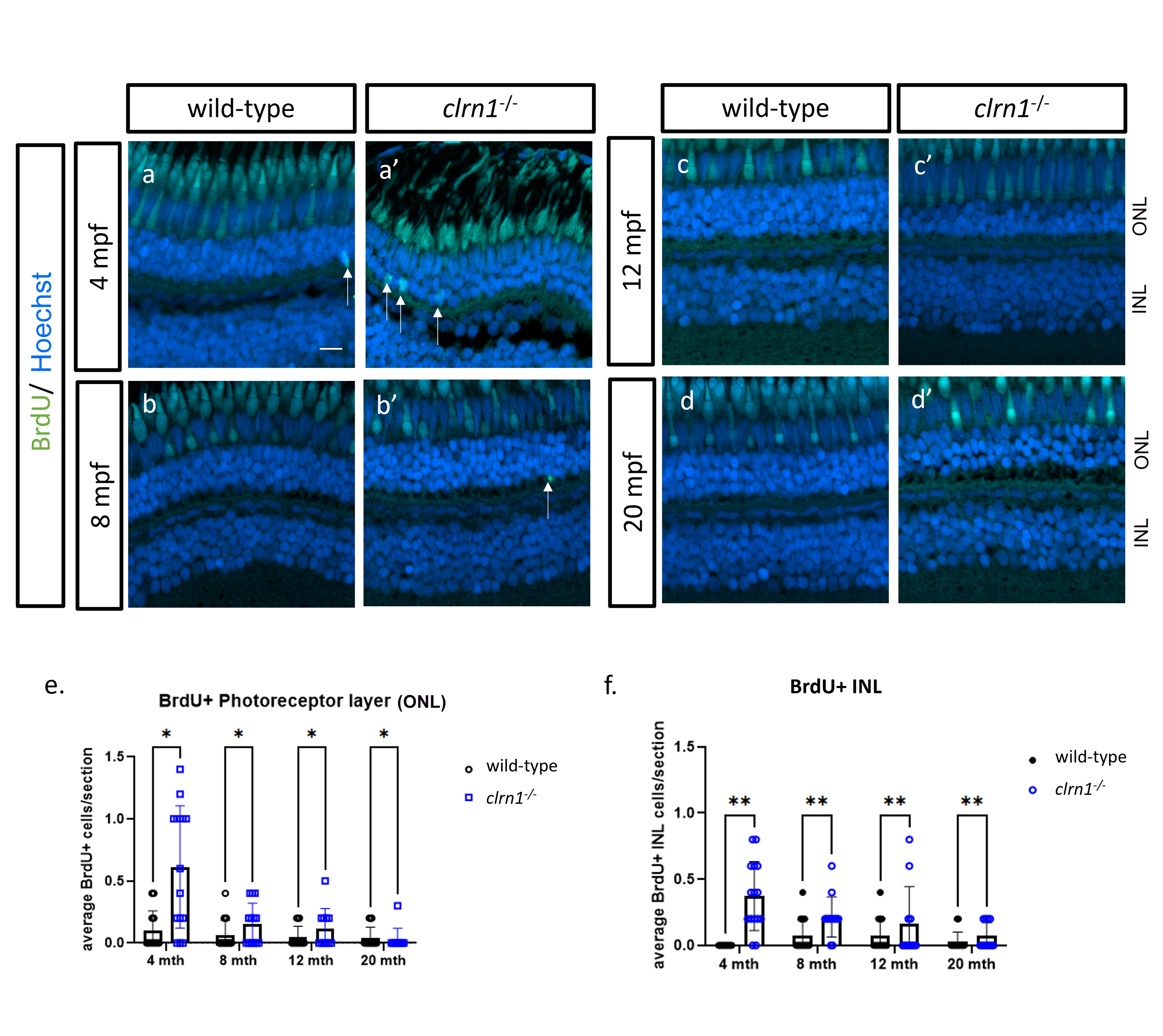

Supplement: S5 Fig — Anti-BrdU (green) staining in paraffin sections from (a,a’) 4 mpf, (b,b’) 8 mpf, (c,c’) 12 mpf, and (d,d’) 20 mpf in wild-type and clrn1-/- zebrafish retinas. Quantification of Brdu+ nuclei in the (e) outer nuclear layer (ONL, or Photoreceptor layer) and (f) inner nuclear layer (INL) revealed an increase in BrdU incorporation for clrn1-/- zebrafish at the youngest time point which decreased with age. White arrows highlight BrdU+ nuclei. (*p<0.05; **p<0.01; One-way ANOVA). Scale bar=10 µm. Data points are averages of 3 sections per retina. n=10 retina per genotype for each time-point. (TIFF) [file pgen.1011205.s005.tif]

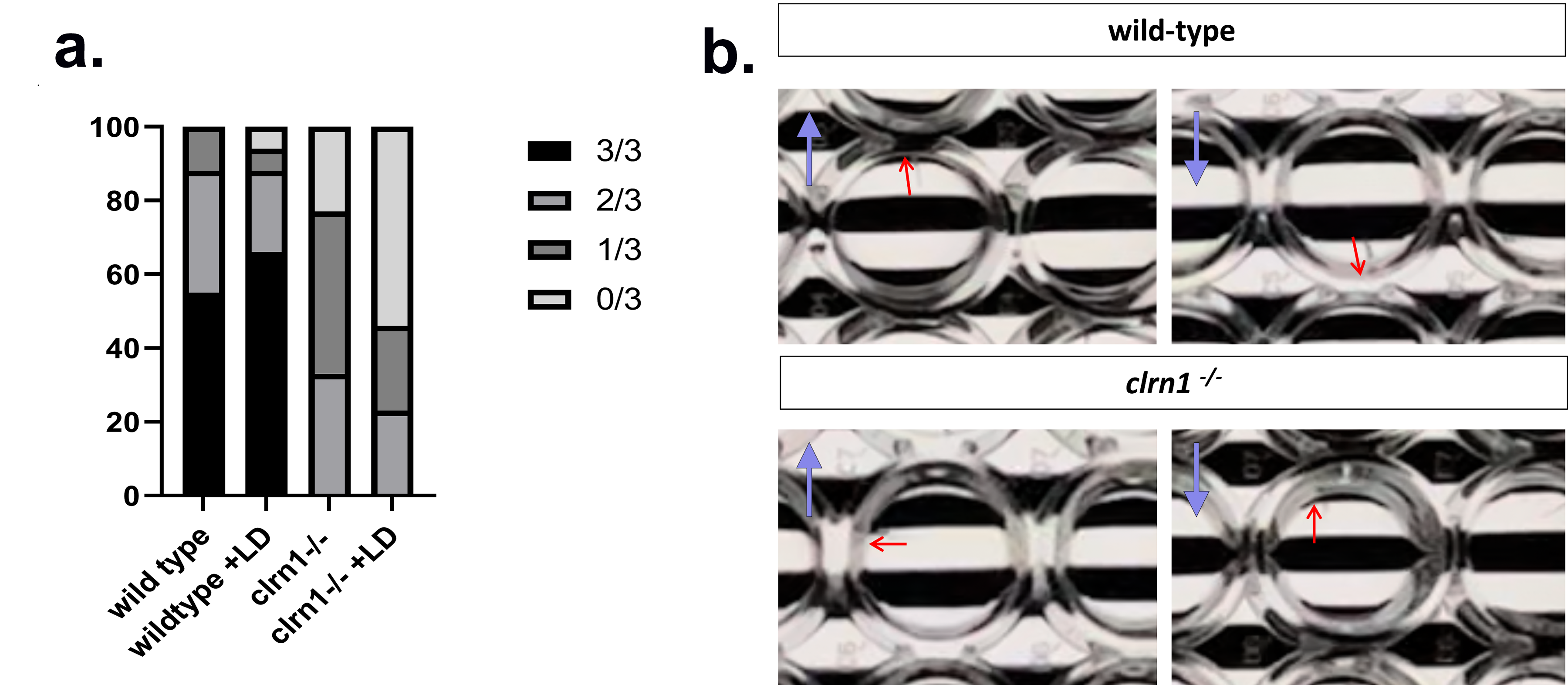

Supplement: S6 Fig — (a) Distribution of OMR responses (number of positive movements in response to 3 direction changes of the moving striped pattern) in wild-type and clrn1-/- control or with light stress (+LS) zebrafish. (b) Representative images of wild-type (upper) and clrn1-/- (lower) larva positions (red arrows) following stimulus direction change (blue arrows indicate direction of moving stripes). (TIFF) [file pgen.1011205.s006.tif]

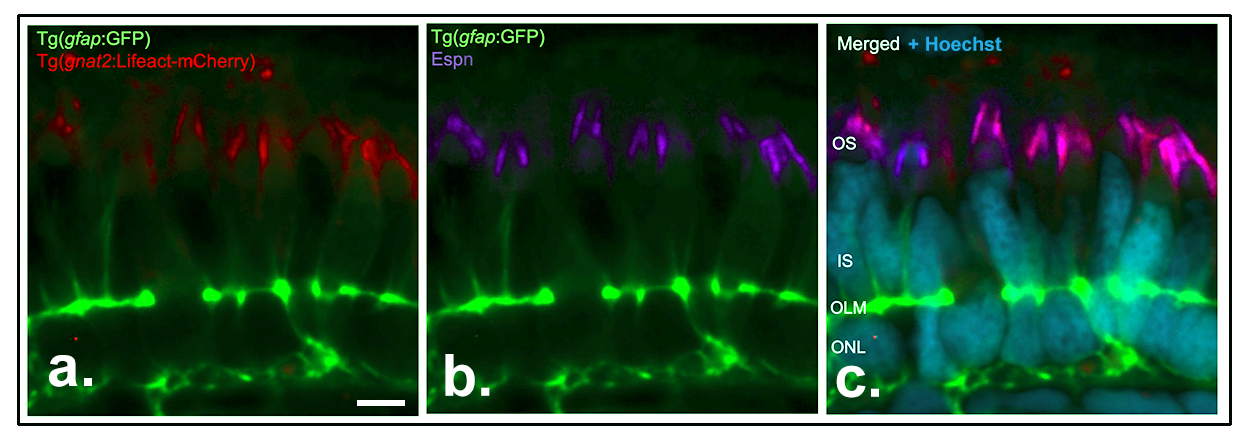

Supplement: S7 Fig — Wild-type 7 dpf larvae were used to investigate whether enriched signal from (a) cone photoreceptor specific Lifeact-mCherry (red) is from calyceal processes marked by (b) Espn immunoreactivity (magenta). (c) Merged images demonstrate significant overlap. Müller glia processes from Tg(gfap:gfp) are shown in green for each image. Hoechst staining (blue) in (c) labels photoreceptor nuclei. Scale bar = 5 µm. OS, Outer Segment; IS, Inner Segment; OLM, Outer limiting membrane; ONL, Outer nuclear layer. (TIFF) [file pgen.1011205.s007.tif]

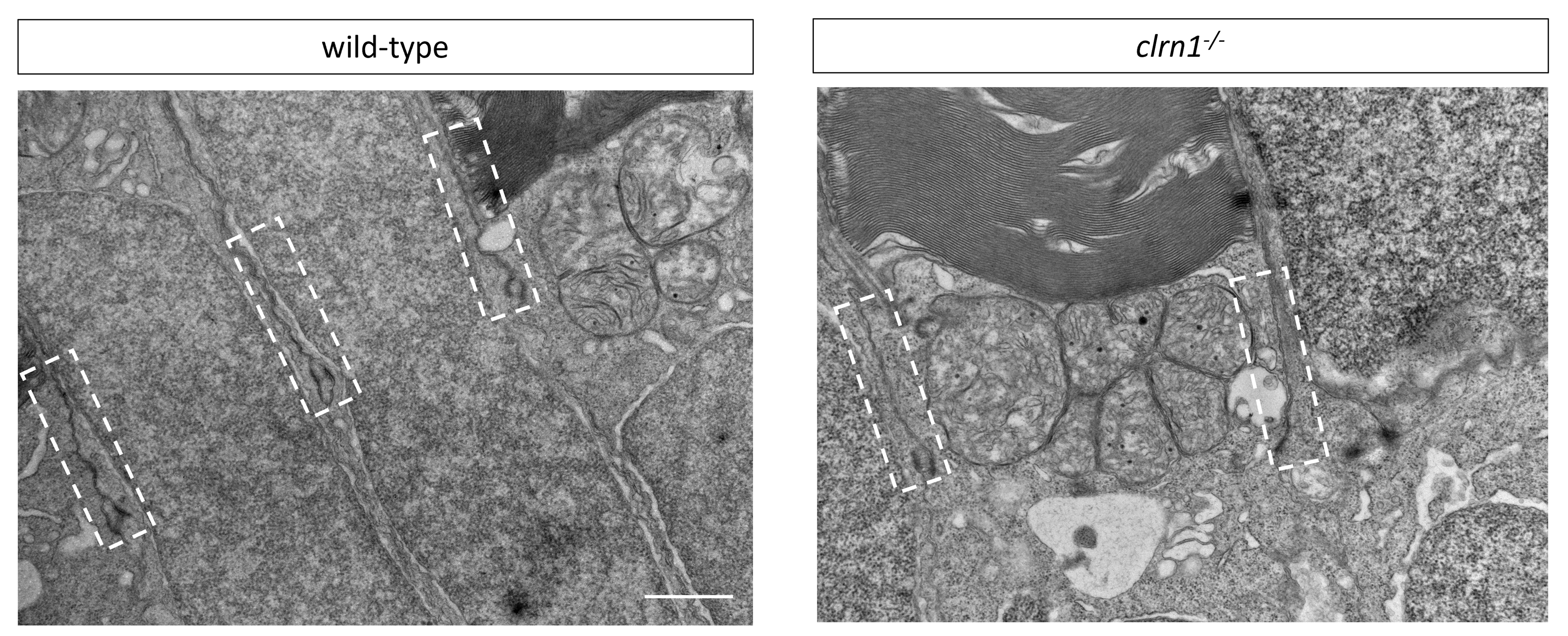

Supplement: S8 Fig — Representative TEM micrographs of the photoreceptor junctions for wild-type and clrn1-/- at 7dpf. White Boxes highlight cell junctions in the OLM. Scale Bar = 1 mm. (TIFF) [file pgen.1011205.s008.tif]

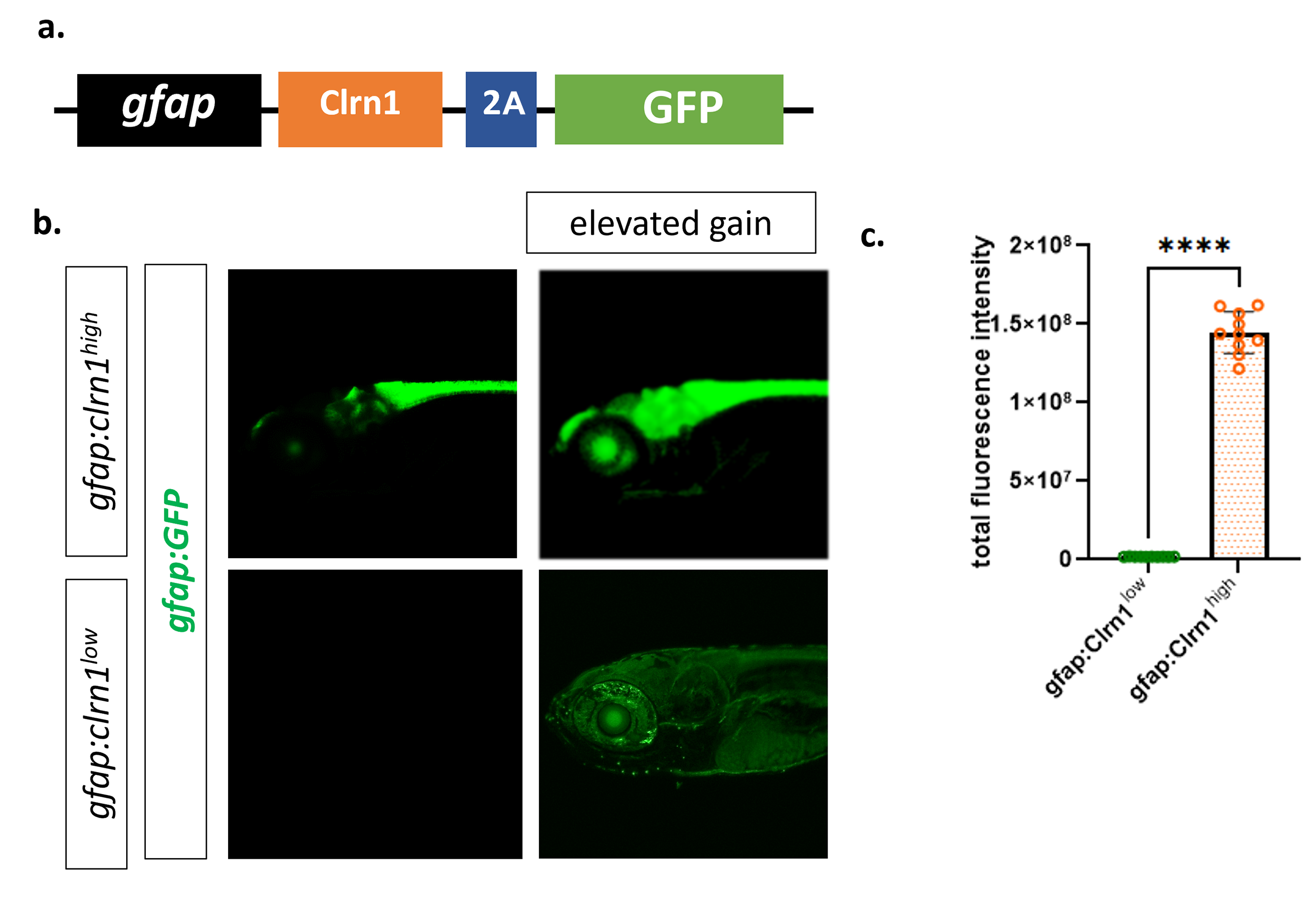

Supplement: S9 Fig — (a) Diagram of Müller glia specific Clrn1 expression transgene (b) Images of the Tg(gfap:Clrn1low) and Tg(gfap:Clrn1high) at a lower and a higher gain to show the low-level reporter expression in the gfap:Clrn1low line. (c). Quantification of total GFP fluorescence intensity in the Tg(gfap:Clrn1low) and Tg(gfap:Clrn1high) lines within the retina. (****p<0.001; Unpaired Students T-test). n=10 zebrafish for each transgenic line. (TIFF) [file pgen.1011205.s009.tif]

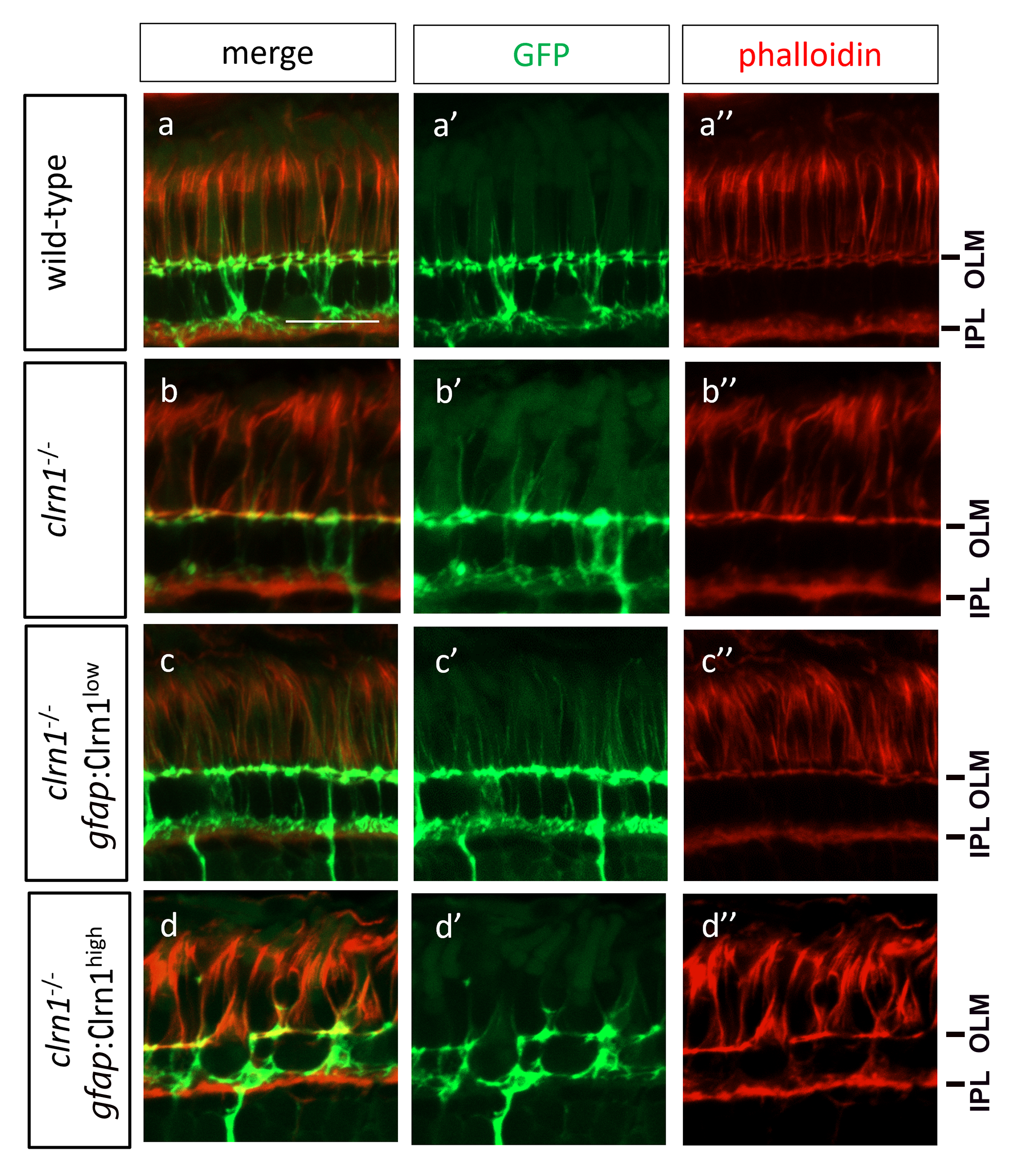

Supplement: S10 Fig — Comparison of Müller glia morphology and actin organization within the outer retina in (a) wild-type, (a) clrn1-/-mutants alone, (c), clrn1-/- mutants with Tg(gfap:Clrn1low), and (d) clrn1-/- mutants with Tg(gfap:Clrn1high). Comparisons reveal that re-expression Clrn1 in clrn1-/- mutants with the Tg(gfap:Clrn1low) transgene corrects disorganization of apical microvilli projections and actin in the outer retina, while re-expression Clrn1 in clrn1-/- mutants with the Tg(gfap:Clrn1high) transgene exacerbates disorganization of these structures. Scale Bar = 50 µm. OLM, Outer Limiting Membrane; IPL, Inner Plexiform Layer. n=10-15 zebrafish per group. (TIFF) [file pgen.1011205.s010.tif]
